# French version of the Rating Scale for aggressive behaviour in the Elderly (F-RAGE): psychometric properties and diagnostic accuracy

**DOI:** 10.1590/S1980-57642013DN70300008

**Published:** 2013

**Authors:** Barry Adama, Calvet Benjamin, Clément Jean-Pierre, Druet-Cabanac Miche, Annie Prado-Jean

**Affiliations:** 1 CMRR. Limousin County Memory Center for Resource and Research, Hospital Center Esquirol of Limoges, France.; 2 Center of Occupational Medicine, University Hospital Center, Limoges, France.; 3 INSERM UMR1094, Tropical Neuroepidemiology, Limoges, France.; 4 University of Limoges, School of Medicine, Institute of Neuroepidemiology and Tropical Neurology, CNRS FR 3503 GEIST, Limoges, France.

**Keywords:** aggressive behaviour, psychogeriatric, RAGE, CMAI, NPI

## Abstract

Aggressive behaviour is the most disturbing and distressing behaviour displayed by elderly people. The prevalence of aggressive behaviour is around 50% among psychogeriatric patients.

**OBJECTIVE:**

This study sought to analyze the psychometric properties and diagnostic accuracy of the French version of the Rating Scale for Aggressive Behaviour in the Elderly (F-RAGE).

**METHODS:**

The F-RAGE was administered to 79 patients hospitalized in a geriatric psychiatry department. A psychiatrist, who was blind to the subjects' RAGE scores, performed the diagnosis for aggressivity based on global clinical impression. The F-RAGE and MMSE were applied by a trained researcher blind to subjects' clinical diagnoses while the Cohen-Mansfield Agitation Inventory and Neuropsychiatric Inventory were administered by medical and nursing staff. Internal consistency, reliability, cut-off points, sensitivity and specificity for F-RAGE were estimated.

**RESULTS:**

F-RAGE showed satisfactory validity and reliability measurements. Regarding reliability, Cronbach's α coefficient was satisfactory with a value of 0.758. For diagnostic accuracy, a cut-off point of 8 points (sensitivity=74.19%; specificity=97.98%) and area under curve of 0.960 were estimated to distinguish between aggressive patients and control subjects.

**DISCUSSION:**

F-RAGE showed acceptable psychometric properties, supported by evidence of validity and reliability for its use in the diagnosis of aggressive behaviour in elderly.

## Abstract (pt)

O comportamento agressivo é o comportamento mais perturbador e angustiante que possa ser apresentado pelos idosos. A prevalência de comportamento agressivo é cerca de 50% entre os pacientes psicogeriátricos.

**OBJETIVO:**

Analisar as propriedades psicométricas e acurácia diagnóstica da versão francesa da Escala de Avaliação do Comportamento Agressivo em Idosos (F-RAGE).

**MÉTODOS:**

A F-RAGE foi administrada a 79 pacientes internados no departamento de psiquiatria geriátrica. Um psiquiatra que era cego às pontuações F-RAGE dos sujeitos realizou o diagnóstico de DSM-IV com base na impressão clínica global. O F-RAGE e MMSE foram realizados por um pesquisador treinado cego ao diagnóstico clínico dos sujeitos e o Inventário de agitação de Cohen-Mansfield e o Inventário Neuropsiquiátrico pela equipe médica e de enfermagem. Consistência interna, pontos de corte, sensibilidade e especificidade para F-RAGE foram estimados.

**RESULTADOS:**

F-RAGE mostrou validade satisfatória e medidas de confiabilidade. Em relação à confiabilidade, coeficiente α de Cronbach foi satisfatória com um valor de 0,758. Para maior precisão de diagnóstico, um ponto de corte de 8 pontos (sensibilidade=74,2%, especificidade=98,0%) e área sob a curva de 0,960 foram estimados para distinguir entre os pacientes agressivos e controles.

**DISCUSSÃO:**

F-RAGE mostrou propriedades psicométricas aceitáveis, apoiados por evidências de validade e confiabilidade para sua utilização no diagnóstico do comportamento agressivo em idosos.

## INTRODUCTION

Aggressive behaviour (AB) is the most disturbing and distressing behaviour displayed by older patients in long-term care facilities or in psychogeriatric units. Patel and Hope^1^ defined AB as an overt act, involving the delivery of noxious stimuli to (but not necessarily aimed at) another object, organism or self, which is clearly not accidental. It affects older patients themselves and their informal and formal caregivers. It can also lead to increased health care costs. Family members and friends are affected by aggressive behaviours in long-term care facilities. They can be embarrassed by these disruptive behaviours and can reduce the frequency of their visits.^2^ Some individual and environmental factors are implicated in the triggering of AB. Dementia is the leading cause of disruptive behaviours and Alzheimer's disease accounts for 60%-80% of such cases in the elderly.^3^ Male gender and being in the younger strata of the older adult population are individual factors associated with AB. Psychiatric diseases such as depression, schizophrenia, anxiety, hallucination and delusion have also been linked to AB.^2,5^

The prevalence of AB varies widely from 7% to 91% in long-term care facilities^4^ and it is estimated to average 50% in psychogeriatric facilities.^5,6^ Patel and Hope found nearly half the sample was at least mildly aggressive over a 3-day period. Rabinset al.^7^ reported a prevalence of 47% in a psychogeriatric ward. In an institution, Zimmeret al.^8^ found that 22.6% of residents had serious behavioral problems. Two-thirds of these patients were diagnosed as suffering from dementia. Prevalence of AB is more significant among community-based patients with dementia. Between 20% and 50% of families of patients with Alzheimer's disease reported AB. Ryden^9^ reported a prevalence of verbal aggression of 49%, physical aggression of 46% and sexual aggression of 17%.

Several tools are used for the assessment of behavioural symptoms, but most of these scales are not specific for one behavioural disorder such as aggression. In addition, they were initially developed for the assessment of behavioural problems in dementia whereas they are not adapted for the measure of these disorders in patients with other psychiatric problems. The Rating Scale for Aggressive Behaviour in the Elderly (RAGE) is a rating scale specifically developed for the assessment of aggressive behaviour in institutionalized or hospitalized elderly^1^. This scale takes into account different dimensions of aggression: verbal and physical aggressions. It has the advantage of being used for different diseases found in psychogeriatric departments, not just for dementia. This study sought to analyze the psychometric properties and diagnostic accuracy of the French version of the Rating Scale for Aggressive Behaviour in the Elderly (F-RAGE) among patients hospitalized in psychogeriatric departments, and compares the diagnostic performances of the F-RAGE to that of the Cohen-Mansfield Agitation Inventory (CMAI).^10,11^

## MATERIALS AND METHODS

**Subjects.** The study was carried out at the Hospital Center Esquirol in 2013 and included participants of both sexes, aged 65 or older, who were hospitalized for at least seven days. Of the 170 patients hospitalized during the inclusion period, only 79 (46.5% of the total population) were enrolled. Patients with acute somatic illness or who presented incapacity to communicate were not included in this study. Patients who were included gave their informed consent (or proxy consent for patients with severe cognitive impairment (MMSE<15)) and underwent a standardized assessment that included taking of a detailed socio-demographic history, and assessments of neuropsychiatric symptoms, aggressive behaviour and cognition. The protocol was approved by the regional board of medical research ethics.

**Assessment.** *Aggressive behaviour –* The gold standard was the diagnosis based on the expertise and global clinical impression of the psychiatrist's blind assessment of other aggressive behaviour scales such as the F-RAGE, the CMAI or the NPI. Based on this expertise, the psychiatrist overseeing the patient stated whether the patient had exhibited verbal or physical aggressive behaviour.

Two instruments were chosen for assessment of aggressive behaviours:

– The French version of the Rating Scale for Aggressive Behaviour (F-RAGE)^1^, for measuring aggressive behaviour in psychogeriatric inpatients. It is designed to be completed by the staff, who are asked to specify the types of aggressive behavior observed in patients under their care for 3 days. The original version of the RAGE was a 21-item scale. Seventeen items concerned specific kinds of aggressive behaviour. Three items enquired about the consequences of the aggressive behaviour, and the final item asked the rater to provide an overall assessment of aggressive behaviour. Each item was rated on a four-point scale based on frequency ranging from 0 (never) to 3 (always). The RAGE was translated into French and back-translated into English with satisfactory pilot testing. The required time for completing the F-RAGE was only about five minutes.
– The French version of the Cohen-Mansfield Agitation Inventory (CMAI)^12^, for comparing the diagnostic performance of the CMAI with the F-RAGE. It is widely used in psychogeriatric units; it measures 29 disruptive behaviors in four dimensions: physical aggression, nonphysical aggression, aggressive verbal and non-verbal aggressive behaviour. In this study, we focused only on two dimensions: physical aggression and aggressive verbal behaviour.

*Cognitive status –* Overall cognitive function was assessed using the Mini-Mental State Examination (MMSE),^13^ a brief 30-point questionnaire test used to screen for cognitive impairment.

*Neuropsychiatric symptoms –* The Neuropsychiatric Inventory (NPI)^14^ is a useful tool for rating the major neuropsychiatric symptoms observed in dementia such as delusion, hallucinations, anxiety, depression, aggressive behaviour/agitation, sleep disorders, eating disorders, apathy, disinhibition, euphoria, aberrant motor activity and irritability.

**Procedures.** This study was conducted in three steps. The first step was the inclusion visit with the psychiatrist who, blind to the subjects' different scores, performed the diagnosis of aggressive behaviour. The last two steps correspond to administration of different rating scales. These last steps were carried out independently and blinded. The order of administration of different rating scale was not pre-defined. Only the assessment of cognitive function was systematically carried out at the inclusion visit. The F-RAGE and MMSE were performed by a trained researcher from the staff team, whereas the NPI was applied by physicians. The CMAI was administered by nursing staff.

**Statistical analysis.** Socio-demographic characteristics of participants were described by frequencies and percentages for qualitative variables and by means and standard deviation for continuous variables. To compare the two groups (aggressive patients and non-aggressive behaviours) on continuous data (age and MMSE, CMAI, F-RAGE, NPI scores), Student's *t* test was used, whereas qualitative variables (sex, marital status) were compared using the Chi-square test. Cronbach's α and split-half correlation coefficients were calculated for internal consistency analysis. For the validity analysis, the mean F-RAGE scores of aggressive and non-aggressive groups were compared using Student's *t* test. The diagnostic performance was assessed by reference to two standard criteria: sensitivity, specificity. This graphic representation allows definition of these performances and choice of optimal cut-off. To compare diagnostic performances between the Cohen-Mansfield Agitation Inventory (CMAI) and the French version of the Rating Scale for Aggressive Behaviour in the Elderly (F-RAGE), areas under curves were compared using the Hanley and McNeil's method. Items were factor analyzed using principal component extraction and orthogonally rotated using Varimax rotation. Following this significant analysis, the items which did not correlate with at least one other variable at a value greater than 0.5, were dropped from the analysis. Factors with eigenvalues >1 were extracted. To compare the diagnostic performances between the two tests, we applied the Hanley and McNeil's method. All analyses were performed using SPSS for Windows 20.0 and ROC analysis conducted with MedCalc^®^ 12.7.0. Level of significance was 0.05 for all analyses.

## RESULTS

**Sample characteristics.** The sample comprised 79 patients with a mean age of 83.3±6.8(66-94) years. The average MMSE score was 17.9±7.1(2-30) (Table 1) and 36 subjects had been diagnosed as suffering from dementia according to DSM-IV-TR. Thirty-one patients were aggressive according to the psychiatrist global clinical impression. The sex ratio (male/female) was 0.58 (29 men and 50 women). All patients could read and write. Fifty patients lived alone or in nursing homes. Among the reasons for hospitalization, 35 were for affective symptoms, 31 were for behaviour disturbances (apathy, agitation, disinhibition), 9 were for psychotic disturbances and 4 were for other reasons (cognitive impairment, bipolar disorders). The score for the Cohen-Mansfield Agitation Inventory (CMAI) total population was 46.2±22.^2^ (28-148), for the French version of the Rating Scale for Aggressive Behaviour in the Elderly (F-RAGE) score was 9.3±13.1(0-9) and for the Neuropsychiatric Inventory (NPI) the score was 16.4±11.6(0-49) (Table 1).

**Table 1:** Socio-demographic characteristics of aggressive and non-aggressive subjects.

	Study populationN=79	Aggressive subjectsn=31	Non-aggressive subjectsn=48	Significancep
Mean age	82.7±8.7	80.2±11.2	84.4±6.2	0.1
Sex (% female)	50 (63.3)	14 (45.2)	36 (75.0)	0.01
Marital status (% living alone)	50 (63.3)	15 (48.4)	35 (72.9)	0.028
MMSE mean±SD	17.9±7.1	15.13±7.8	19.7±5.9	0.009
CMAI mean±SD	46.2±22.1	63.3±25.8	35.2±8.5	<0.001
F-RAGE mean±SD	9.3±13.1	21.7±12.9	1.2±3.1	<0.001
NPI mean±SD	16.4±11.6	22.1±10.9	12.8±10.7	<0.001

MMSE: Mini-Mental State Examination; CMAI: Cohen-Mansfield Agitation Inventory, F RAGE: French version of Rating Scale for Aggressive Behaviour in the Elderly, NPI: Neuropsychiatric Inventory, SD: Standard Deviation.

**Comparison of aggressive and non-aggressive patients.** The population was divided into two groups on the basis of the psychiatrist's global clinical impression. The proportion of females (p=0.010) and of patients living alone (p=0.028) was significantly higher in the group of non-aggressive patients. According to the NPI, affective symptoms were more frequent in non-aggressive group (p=0.001) while behaviour disorders were more frequent in the aggressive group (p=0.010). There was no significant difference in the proportion of dementia between the two groups. MMSE score was significantly higher in the non-aggressive group (p=0.009). There were significant differences in scores for the following NPI dimensions: irritability, aggressive behaviour, anxiety, disinhibition, and aberrant motor activity (Table 2).

**Table 2:** Clinical characteristics of aggressive and non-aggressive subjects.

Mean score±SD		Study populationn=79	Aggressive subjectsn=31	Non-aggressive subjectsn=48	Significancep
Neuropsychiatric Inventory	Hallucinations	1.0±2.4	1.9±3.4	0.4±1.1	0.09
	Delusions	1.7±3.2	1.8±3.6	1.6±3.0	0.7
	Aggression/agitation	2.7±4.0	6.7±3.9	0.2±0.7	<0.001
	Depression/dysphoria	2.9±3.8	2.3±3.8	3.3±3.9	0.19
	Anxiety	1.7±2.6	0.7±1.4	2.4±3.0	0.004
	Euphoria	0.3±1.5	0.1±0.4	0.4±1.9	0.53
	Apathy	1.2±3.0	1.4±3.6	1.1±2.6	0.7
	Disinhibition	0.7±2.2	1.5±3.3	0.2±0.6	0.01
	Irritability	0.9±2.1	2.2±3.0	0.1±0.5	0.03
	Aberrant motor activity	1.0±2.6	1.8±3.6	0.5±1.6	<0.001
	Sleep disorders	1.3±2.5	0.9±1.7	1.5±2.9	0.44
	Eating disorders	1.2±2.6	1.0±2.1	1.3±2.9	0.71
	Total score	16.4±11.6	22.1±10.9	12.8±10.7	<0.001
CMAI	Non-aggressive physical behaviour	21.8±11.8	29.9±14.6	16.9±5.6	<0.001
	Non-aggressive verbal behaviour	6.9±4.1	8.5±4.6	5.9±3.3	0.003
	Aggressive physical behaviour	12.2±7.9	17.1±11.0	9.1±0.9	<0.001
	Aggressive verbal behaviour	5.4±3.8	8.4±3.9	3.5±2.0	<0.001
	Total Score of aggression	17.7±10.7	25.5±13.7	12.6±2.3	<0.001
	Total Score	46.2±22.1	63.3±25.8	35.2±8.5	<0.001
F-RAGE	Total Score	9.3±3.1	21.7±12.9	1.2±3.1	<0.001

CMAI: Cohen-Mansfield Agitation Inventory, F-RAGE: French version of the Rating Scale for Aggressive Behaviour in the Elderly; SD: Standard deviation.

Selection of items. In order to optimize the Cronbach's α reliability index, we decided to eliminate items 12 and 21 in agreement with psychiatrists. Question 12 of the F-RAGE was also dropped because of its redundancy with the question 17. Question 21 was deleted because it summarizes the global measure of aggressive behaviour. Logically the total rating score should determine the presence of aggressive behaviour and the magnitude of this behaviour disorder. The principal component analysis was applied in order to delete those items which did not have a correlation coefficient greater than 0.5. Subsequently, item 1 was deleted for this reason. Finally, we kept a construct of 18 items. Its total score was 52 whereas the original version of the RAGE was 61.

**Factor structure.** Five factors with eigenvalue >1 were extracted and these accounted for 75.1% of the variance. Factor I accounted for 31.4% and reflected mainly physical aggression, factor II accounted for 16.4% and corresponded to verbal aggression, factor III accounted for 11.1%, reflecting self-destruction, factor IV accounted for 8.5% and reflected the possible consequence of aggressive behaviour. Factor V accounted for 7.7% and related to antisocial acts (Table 3).

**Table 3:** Factor analysis of items of the RAGE after Varimax rotation.

Items	FactorI	Factor II	Factor III	Factor IV	Factor V
Item 15	0.947				
Item 14	0.919				
Item 11	0.895				
Item 16	0.768				
Item 5	0.561				
Item 7		0.837			
Item 9		0.727			
Item 3		0.720			
Item 8		0.681			
Item 6		0.613			
Item 10			0.889		
Item 13			0.850		
Item 18			0.784		
Item 19			0.715		
Item 17				0.708	
Item 20				0.678	
Item 2				0.553	
Item 4					0.865
Item 1					**0.457**
Eigenvalue	5.974	3.108	2.106	1.616	1.462
Percentage of variance	31.4	16.4	11.1	8.5	7.7

**Figure 1:**
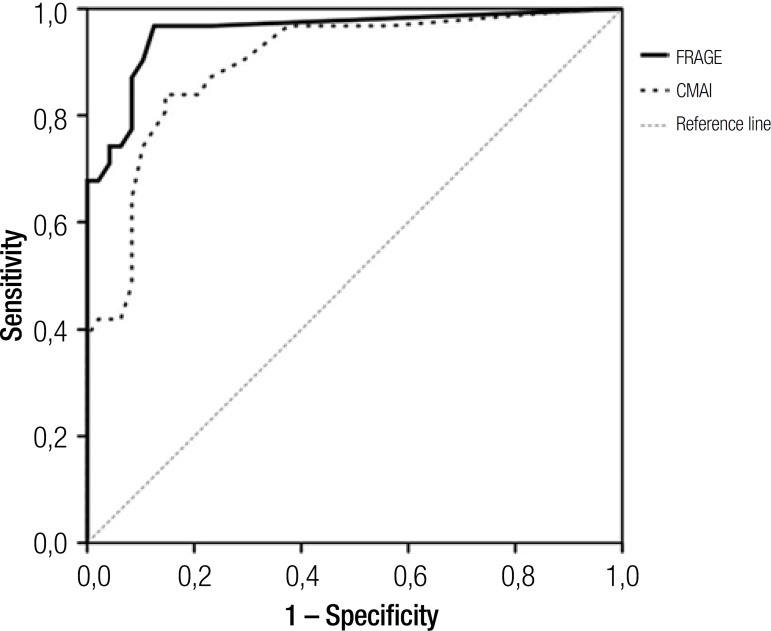
Receiver operating characteristic (ROC) analysis: comparison of the French version of the Rating Scale for Aggressive Behaviour in the Elderly (F-RAGE) and the Cohen-Mansfield Agitation Inventory (CMAI) with diagnosis of aggression by psychiatrist clinical diagnosis.

**Determination of RAGE diagnostic performance.** Cronbach's α was 0.76 and the split-half correlation coefficient was 0.74. Validity analysis showed that the aggressive group had a significantly higher mean F-RAGE total score than the group without aggression (21.7±2.3v 1.2±0.5, P<0.001). The highest sum of sensitivity and specificity values, 172.1, was obtained for the cut-off score of 8/9. Sensitivity, specificity, positive predictive value (PPV), and negative predictive value (NPV) for cut-off scores between 1 and 40 are shown in Table 4. The ROC curve also showed that the 8/9 provided the best results, since they were very close to each other, with 8/9 being closest to the upper left of the graph. The area under the curve (AUC) value was 0.96±0.03(95% CI 0.89 to 0.99; P<0.001). A sensitivity value of 74.2 95% CI (55.4-88.1) and a specificity of 97.9 95% CI (88.5-99.9) were determined. In this population, the prevalence of aggressive behaviour was 39.2%. We found a positive predictive value of 95.8% 95% CI (78.3-99.9) and a negative predictive value of 85.6 95% CI (73.5-93.6) .The final test of the French version of the Rating Scale for Aggressive Behaviour in the Elderly (RAGE) is illustrated by the receiver operating characteristic curve depicted in Figure 1.

**Table 4:** Discrimination between aggressive (n=31) and non-aggressive (n=48) groups.

Cut-off	Sensitivity	95% CI	Specificity	95% CI	PPV	95% CI	NPV	95% CI
0	100.0	88.8-100.0	0.0	0.0-7.4	39.0	28.2-50.6		
0/1	96.8	83.3-99.9	75.1	62.7-88.0	73.0	56.6-85.8	97.4	86.2-99.9
1/3	93.6	78.6-99.2	89.6	77.3-96.5	85.2	68.5-95.1	95.6	84.9-99.5
3/4	90.3	74.2-98.0	89.6	77.3-96.5	84.7	67.6-94.9	93.5	82.2-98.7
4/5	77.4	58.9-90.4	91.7	80.0-97.7	85.6	66.8-96.0	86.4	73.9-94.4
5/6	74.2	55.4-88.1	91.7	80.0-97.7	85.1	65.7-95.9	84.7	72.1-93.2
6/7	74.2	55.4-88.1	93.8	82.8-98.7	88.4	69.2-97.6	85.0	72.6-93.3
7/8	74.2	55.4-88.1	95.8	85.7-99.5	91.9	73.4-99.1	85.3	73.0-93.5
8/9	74.2	55.4- 88.1	97.9	88.9- 99.9	95.8	78.3- 99.9	85.6	73.5- 93.6
9/10	71.0	52.0-85.8	97.9	88.9-99.9	95.6	77.4-99.9	84.1	71.8-92.5
10/11	71.0	52.0-85.8	100.0	92.6-100.0	100	83.9-100.0	84.3	72.3-92.6
11/12	64.5	45.4-80.8	100.0	92.6-100.0	100	82.4-100.0	81.5	69.3-90.4
12/13	61.3	42.2-78.2	100.0	92.6-100.0	100	81.5-100.0	80.2	67.9-89.3
13/14	54.8	36.0-72.7	100.0	92.6-100.0	100	79.4-100.0	77.6	65.2-87.2
15/17	45.2	27.3-64.0	100.0	92.6-100.0	100	75.3-100.0	74.0	61.7-84.1
19/20	41.9	24.5-60.9	100.0	92.6-100.0	100	73.5-100.0	72.9	60.6-83.1
10/11	38.7	21.8-57.8	100.0	92.6-100.0	100	71.5-100.0	71.8	59.5-82.2
22/25	32.3	16.7-51.4	100.0	92.6-100.0	100	66.4-100.0	69.8	57.5-80.3
25/26	25.8	11.9-44.6	100.0	92.6-100.0	100	59.0-100.0	67.8	55.7-78.4
13/14	22.6	9.6-41.1	100.0	92.6-100.0	100	54.1-100.0	66.9	54.8-77.5
14/15	19.4	7.5-37.5	100.0	92.6-100.0	100	47.8-100.0	66	54.0-76.7
30/31	16.1	5.5-33.7	100.0	92.6-100.0	100	39.8-100.0	65.1	53.1-75.8
31/38	6.5	0.8-21.4	100.0	92.6-100.0	100	2.5-100.0	62.6	50.8-73.3
38/39	3.2	0.08-16.7	100.0	92.6-100.0	100	0.0-100.0	61.8	50.1-72.6
39/40	0.0	0.0-11.2	100.0	92.6-100.0			61	49.4-71.8

CI: confidence interval. NPV: negative predictive value. PPV: positive predictive value. cut-off calculated on 18 items.

**Comparison of F-RAGE versus cmAi diagnostic performance.** To compare F-RAGE with CMAI performance, areas under curve of two graphic representations were used. The area under curve of CMAI was 0.894±0.040(95% CI 0.81 to 0.95). The difference in areas was 0.058±0.042(CI 95%: –0.024; 0.142) but this difference was not significant (p=0.166). The two tests are illustrated by the Receiver Operating Characteristics presented in Figure 1.

## DISCUSSION

Our results suggested that the French version of the RAGE (F-RAGE) is a valid instrument for measuring aggressive behaviour (AB) in French elderly. The F-RAGE is a useful tool for rating these behavioral disorders in psychogeriatric demented and non-demented inpatients and can be easily used by nursing staff in routine procedures. Moreover, completing the questionnaire takes only around ten minutes.

In this sample, aggressive inpatients were more likely to be of male gender and have greater cognitive decline than non-aggressive inpatients. Aggressive inpatients also more often had other behavioral or psychological disorders compared to nonaggressive subjects. All these socio-demographic characteristics and clinical results are consistent with other international studies.^15-17^ Aggressive behaviour was reported in 39% of inpatients. This prevalence is lower than those reported in the literature.^5-B7^ Several studies found that nearly half of the samples exhibited aggression. These differences could be due to the variability of tools used, the definition used for aggressive behaviour, and the percentage of demented patients. In fact, dementia is a common etiology of aggressive behaviours in the elderly.^18-20^ In the present study, 46% of inpatients suffered from dementia. However, this percentage is lower than the rate found for example in nursing homes or long-term care facilities.^2,21-23^

In our study, we chose the clinical diagnosis established by psychiatrists, in consultation with the health care team of the psychogeriatric ward, as the gold standard. This observational method for the diagnosis of these disorders is frequently used in the international literature.^24,25^

In the original version of the RAGE, the Cronbach's α was 0.89. We found a satisfactory value (0.758) which exceeded the permitted acceptability threshold in the scientific community.^26^ During the validation of the Chinese version of the RAGE, Lamet al.^6^ found a coefficient of 0.74. These results are in accordance with those for F-RAGE. The diagnostic performance of the French version of the RAGE was evaluated by sensitivity (74%), specificity (98%), and positive and negative predictive values (96% and 86%, respectively). For the F-RAGE, we chose a cutoff ≥8 because it demonstrated the combination of the highest sensitivity and specificity for this version. In the original and the Chinese version of the RAGE, there is no mention of any level of specificity or sensitivity based on the overall score or specific score for each dimension of aggression.^1,6^

We observed no significant difference in diagnostic performance between the F-RAGE and the CMAI. Although this result was not significant, it is clear that the F-RAGE measures more dimensions of aggressive behaviour than the CMAI which primarily evaluates agitation. The F-RAGE is thus more adapted to take account of all dimensions of aggression in elderly. The small size of our sample and the high Pearson r correlation between these two scales (r=0.73)^1^ might explain why we did not observe a significant difference, but this was not the main objective of this study.

The management of AB in the elderly may require physical and pharmacological approaches such as medications which are associated with several adverse effects.^27-31^ Moreover, these approaches, when used in a non-rational way, can harm the individual's dignity and leave the patient prone to damaging side effects, and even the risk of abuse.^32^ Neuroleptics are often used to control physical and verbal aggressions whereas benzodiazepines are more often employed to reduce verbal aggressive behaviour.^4,33,34^ Physical restraint is used in an attempt to control aggressive or other risky behaviors. This approach can lead to serious adverse effects for patient health, such as loss of autonomy and self-esteem, and worsening of AB or disruptive behaviour.^35-39^ Another significant finding is that professional carers experience considerable stress, negative feelings and burnout as a result of being the victims of AB in institutions.^40-44^ Consequently, having a valid instrument for identifying aggression is very important both for the health care team and patients.

According to the principal component analysis, the F-RAGE is composed of five dimensions of AB as the original scale: physical aggressive behaviour, verbal aggressive behaviour, antisocial acts, self-harming and consequences of aggressive behaviour. Self-harming is the main reason for use of physical restraint in institutions or in psychogeriatric wards.^36,39^ The antisocial dimension is relevant to study because it produces frustration, emotional distress, increasing absenteeism and burn-out of carers and leads to violence against patients by carers. Finally, consequences of aggressive behavior are a dimension which leads to use of drugs in order to reduce these behavioral disorders. Identifying these different dimensions allows more suitable management for each type of aggressive behaviour in institutions or care units. Our study provides the validation of the French version of the RAGE. However, further studies are needed to verify the inter-rater and test-retest reliabilities of this French version.
